# Protective Effect of Hydroxytyrosol Against Oxidative Stress Induced by the Ochratoxin in Kidney Cells: *in vitro* and *in vivo* Study

**DOI:** 10.3389/fvets.2020.00136

**Published:** 2020-03-31

**Authors:** Rosalia Crupi, Ernesto Palma, Rosalba Siracusa, Roberta Fusco, Enrico Gugliandolo, Marika Cordaro, Daniela Impellizzeri, Carmen De Caro, Luigino Calzetta, Salvatore Cuzzocrea, Rosanna Di Paola

**Affiliations:** ^1^Department of Veterinary Science, University of Messina, Messina, Italy; ^2^Department of Health Sciences, University of Catanzaro “Magna Graecia”, Catanzaro, Italy; ^3^Department of Chemical, Biological, Pharmaceutical and Environmental Sciences, University of Messina, Messina, Italy; ^4^Department of Experimental Medicine, University of Rome “Tor Vergata”, Rome, Italy; ^5^School of Medicine, St. Louis, Mo, United States

**Keywords:** canine kidney cell line, ochratoxin, pig kidney cell line, rabbit kidney cell line, rats

## Abstract

Ochratoxin-A (OTA) is a mycotoxin that is a common contaminant of food products for both humans and animals. This mycotoxin has several toxic effects. In particular, ochratoxin has significant nephrotoxic potential. In fact, OTA has been described as being responsible for naturally occurring animal and human kidney disorders. The toxicity of this mycotoxin involves the induction of the oxidative stress pathways. Therefore, in the present study, we wanted to evaluate the potential protective effects of hydroxytyrosol (HT), a phenolic constituent with potent antioxidant activity, of extra virgin olive oil in three different renal cell lines, the Madin-Darby canine kidney cell line (MDCK), a pig kidney cell line (LLC-PK1), and a rabbit kidney cell line (RK 13), and in rats. Our results clearly showed that renal cells respond to OTA exposure by reducing cell proliferation and the induction of oxidative stress. Pre-incubation of the cells with HT prevented the cellular cytotoxicity and increased reactive oxygen species (ROS) levels induced by OTA. In addition, the antioxidative activity of HT was studied by measuring malondialdehyde (MDA) and lactate dehydrogenase (LDH) levels and nitrosative stress. Finally, we investigated the capability of HT (20 mg/kg, intraperitoneally) to act *in vivo*. In rats, HT reduced oxidative stress and collagen accumulation in the kidney and counteracted the augmentations in AST, ALT, and creatinine levels following OTA induction (250 μg/kg for 90 days orally). In conclusion, our findings demonstrate that HT is able to protect three renal cell lines from the damage induced by OTA and protect the kidneys of rats. Therefore, the use of this compound could be an important strategy for the treatment and prevention of this type of kidney dysfunction.

## Introduction

Mycotoxins are natural products characterized by a low molecular weight that are made as secondary metabolites by filamentous molds or fungi. Under appropriate humidity and temperature conditions, these may accumulate in different feeds and foods, causing serious risks to animal and human health (1). More than 300 mycotoxins have been isolated so far, though most of them are not well-known because of a lack of basic knowledge regarding their toxicity and toxicokinetics. Examples of mycotoxins of the utmost public health and agro-economic importance include fumonisins (Fs), trichothecenes, ochratoxins (OTs), zearalenone (ZEN), and aflatoxins (AFs) (2). These toxins represent a serious worldwide issue in terms of animal and human health and condemned agricultural products (3). Ochratoxin-A (OTA) is a secondary metabolite formed by numerous species of Penicillium and Aspergillus. Numerous pathologies related to mycotoxin exposure in animals and humans have been demonstrated, and thus, mycotoxins have become a global issue (1, 4, 5). Tissue spreading after exposure of animals to OTA has regularly been discovered to be to the highest concentrations in the kidneys and then in either liver or muscle. Other tissues found to contain OTA are the skin, gastric mucosa, myocardium, bone marrow, adrenal medulla, and cortex (6). Numerous studies have highlighted the involvement of oxidative stress in the toxicity of OTA (7–9). The kidney is an important setting for severe oxidative processes in the body, and reactive oxygen species (ROS) play a noteworthy role in the pathogenesis of a diversity of renal disorders (10, 11). Extra virgin olive oil includes minor phenolic constituents that, in addition to giving it its particular aroma and palate, have significant antioxidant effects (12, 13). The principal phenols are hydroxytyrosol (HT), tyrosol (T), and their secoiridoid derivatives. The extraordinary antioxidant activity of HT has stimulated research in numerous fields. Some health-supporting properties have been ascribed to HT, including cardioprotective, antioxidant, antidiabetic, antimicrobial, antitumoural, and neuroprotective activities (14). Current investigations have correspondingly demonstrated beneficial effects of HT on the liver, where olive oil phenols impede the synthesis of cholesterol, triglycerides, and lipids (15, 16). Moreover, it has been described in epidemiological reports that dietary consumption of foods rich in antioxidants, such as phenolic compounds, is linked with a reduced risk of renal dysfunction (17). On the basis of all of this evidence, the objective of our study was to assess the nephrotoxicity effect of OTA-A, using renal cells and rats, to evaluate the protective effect of HT. We performed our research on three different cell lines [LLC-PK1 (pig kidney cell line, proximal tubule epithelial cells), MDCK (Madin-Darby canine kidney cell line, distal tubule epithelial cells), and RK 13 (a rabbit kidney cell line] and in male Sprague–Dawley rats.

## Materials and Methods

### Cell Culture

MDCK, LLC-PK1, and RK were routinely incubated in a humidified atmosphere containing 5% CO_2_ at 37°C. Cells were cultivated in Eagle's MEM culture medium with 10% fetal bovine serum (FBS), 100 IU/mL (60 μg/mL) penicillin, 2.5 μg/mL amphotericin B, and 100 μg/mL streptomycin added.

### Experimental Design

#### First Set of Experiments

1. CTR, cells cultured with standard culture medium;
2. OTA 2.5 μg/mL, cells treated with Ochratoxin (2.5 μg/mL);
3. OTA 5 μg/mL, cells treated with Ochratoxin (5 μg/mL);
4. OTA 15 μg/mL, cells treated with Ochratoxin (15 μg/mL);
5. OTA 25 μg/mL, cells treated with Ochratoxin (25 μg/mL);
6. HT 10 μM, cells treated with Hydroxytyrosol (10 μM);
7. HT 25 μM, cells treated with Hydroxytyrosol (25 μM);
8. HT 50 μM, cells treated with Hydroxytyrosol (50 μM);
9. HT 100 μM, cells treated with Hydroxytyrosol (100 μM);
10. HT 250 μM, cells treated with Hydroxytyrosol (250 μM);
11. HT 500 μM, cells treated with Hydroxytyrosol (500 μM);
12. HT 1,000 μM, cells treated with Hydroxytyrosol (1,000 μM);
13. HT 2,500 μM, cells treated with Hydroxytyrosol (2,500 μM);

#### Second Set of Experiments

1. CTR, cells cultured with normal culture medium;
2. OTA 2.5 μg/mL, cells stimulated with Ochratoxin (2.5 μg/mL);
3. OTA+HT (10 μM): cultures stimulated as described, and HT 10 μM placed in culture medium 30 min before injury.

### Cell Viability Assays

In the first experiment, the cytotoxicities of mycotoxin and HT were evaluated. Samples of 4 × 10^4^ cells were seeded in a volume of 150 μl in 96-well plates. Cells were incubated with culture medium containing increasing concentrations of OTA (2.5, 5, 15, and 25 μg/mL) or HT (10, 25, 50, 100, 250, 500, 1,000, and 2,500 μM) for 24 h. In a second experiment, the effect of HT (10 μM) to reduce the cytotoxicity of OTA was evaluated. Cell viability was measured by MTT assay. Briefly, cells were incubated (at 37°C) with 0.2 mg/mL MTT for 1 h, and then the optical density at 550 nm was read with a microplate reader (OD550).

### Intracellular ROS Determination

Intracellular ROS levels were revealed with an ROS detection kit, as seen previously (18). After several treatments, the kidney cell lines were trypsinized and then washed twice with 1X washing buffer. The cells were incubated with 5-(and-6)-carboxy-2′,7′ -dichlorodihydrofluorescein diacetate (carboxy-H_2_DCFDA; 10 μM final concentration) at 37°C in the dark for 30 min. The fluorescence was read with a microplate reader. The levels of intracellular ROS were expressed as the percentage of the control (nmol/mL).

### Lactate Dehydrogenase (LDH) Assay

The catalytic activity of LDH was calculated by kinematic UV test using 50 μl of culture supernatant, 2 mL of working reagent (61.43 mmol dm^−3^ Tris buffer, 0.20 mmol dm^−3^ NADH, pH = 7.4), and 10 μl of substrate (21.5 mmol dm^−3^ pyruvate). Absorbances were calculated at 339 nm every 30 s throughout a 5 min time period with a UV/Vis spectrophotometer (Pye-Unicam SP8-100).

### Determination of Malondialdehyde (MDA) Levels

Cell-lines MDCK, LLC-PK1, and RK 13 (1 × 10^5^ cells/ well) were seeded in a six-well plate. The cells were used for MDA level measurement using the MDA assay kit, as previously described (19).

### Measurement of Nitrite Levels

Total nitrite levels, as an indicator of nitric oxide (NO) production, were measured in the supernatant as previously described (19). Briefly, nitrate in the medium was reduced to nitrite by incubation with nitrate reductase (670 mU/ml) and β-nicotinamide adenine dinucleotide 3-phosphate (160 mM) at room temperature for 3 h. Entire nitrite concentration was later calculated by performing the Griess reaction by adding 100 μl Griess reagent [0.1% (w/v) N-(1-naphthyl) ethylenediamine dihydrochloride in H_2_O and 1% (w/v) sulfanilamide in 5% (v/v) concentrated H_3_PO_4_; volume 1:1] to the 100-μl sample. OD550 was determined using an enzyme-linked immunosorbent assay microplate reader (Tecan, Männedorf, Switzerland). Nitrite concentrations were measured by comparison with OD550 of standard solutions of sodium nitrite prepared in H_2_O.

### Animals

Male Sprague–Dawley rats (Harlan, Italy) weighing 200–250 g were employed. Animals were divided into groups of two in standard rat cages (Tecniplast s.p.a. VA, Italy) with food and water *ad libitum*. Animals were maintained at 22 ± 1°C with a 12-h light, 12-dark cycle. The University of Messina Review Board for the care of animals permitted the research. All animal experiments observed the new regulations in Italy (D.Lgs 2014/26), as well as EU regulations (EU Directive 2010/63). All procedures were designed to minimize animal suffering. The animals study was conducted according to ARRIVE guidelines.

### Experimental Design

The sample size was estimated using the statistical test a priori power analyses of G-power software. Rats were then randomly divided into four experimental groups of 10 rats each; for each experimental group, the rats were divided into groups of two per cage.

1. Control + vehicle: Each rat received 0.5 mL of saline by oral gavage.
2. Control + HT: Each rat received an intraperitoneal injection (i.p.) of HT at a dose of 20 mg/kg/twice daily (data not shown).
3. OTA group: Each rat received OTA, orally, at a dose of 250 μg/kg for 90 days. OTA was dissolved in 0.1 M NaHCO, pH 7.4, then diluted in 0.5 mL saline, as previously described (20–22).
4. OTA + HT group: Each rat received OTA as described above plus HT by i.p. at a dose of 20 mg/kg/twice daily.

The dose and route of HT administration were based on a previous study by Capasso et al. (23).

### Histological Evaluation

For histopathological studies, at the end of the experiment, the animals were sacrificed by cervical dislocation, and the right kidney was removed, fixed in buffered formaldehyde solution (10% in PBS) at room temperature, dehydrated by graded ethanol, embedded in Paraplast, and cut into 7 μm-thick portions. Tissue portions were deparaffinized with xylene, and then Haematoxylin/Eosin (H&E) staining was performed. Optical microscopy was performed with a Leica DM6 (Milan, Italy) microscope, as previously described by Cordaro et al. (24).

### Masson Trichrome

To assess the grade of fibrosis, kidney sections were stained with Masson trichrome according to the manufacturer's protocol (Bio-Optica, Milan, Italy), as previously described by Di Paola et al. (25).

### Assay of Oxidative Stress

Assay of oxidative stress was performed as previously described (26, 27). MDA level, SOD activity, GSH level, and CAT activity were spectrophotometrically evaluated according to the instructions for assay kits.

### Assessment of Blood AST, ALT, and Creatinine

Blood collected by direct cardiac puncture was allowed to clot, serum was separated (at 2,500 rpm for 15'), and biochemical parameters, namely the serum enzymes aspartate aminotransferase (AST, U/L), alanine aminotransferase (ALT, U/L), and creatinine, were determined on the same day according to the methods described previously (28, 29). The remaining samples were stored at −20°C for future analysis.

### RT-PCR of TGβ-1 and GAPDH

Total RNA was extracted from nearly 100 mg of frozen left renal cortex through a modification of the acid guanidinium thiocyanate-phenolchloroform method (Tri-Reagent; Sigma), as previously described by Gagliano and colleagues (22). The primer used for TGF-β1 was (5′- CACCTGCACAGCTCCAGGCAC 5′-CTTGCGACCCACGTAGTAGACG); the primer used for GAPDH was (5′-ATGGTGAAGGTCGGTGTGAAC - 5′- GCTGACAATCTTGAGGGAGT). The results were normalized to GAPDH gene expression.

### Western Blot Analysis for α-Smooth Muscle Actin (α-sma)

Western blot was performed as previously described for left kidney samples (30), using the following primary antibodies: anti-α-sma (1:1.000; Santa Cruz Biotechnology #sc53142) and anti-β-actin (1:1.000; Santa Cruz Biotechnology).

### Statistical Analysis

All values are shown as mean ± standard error of the mean (SEM) of N observations. N denotes the number of animals employed. In all of the experiments, including immunohistochemistry and histology, at least three experiments were performed. Data were analyzed by one-way ANOVA, followed by a Bonferroni *post-hoc* test for multiple comparisons. A *p* < 0.05 was considered significant. ^*^p < 0.05 vs. Control; #*p* < 0.05 vs. Vehicle.

### Materials

Except where otherwise indicated, all substances were acquired from Sigma-Aldrich (St. Louis, MO, USA). Stock solutions were made in saline (non-pyrogenic, 0.9% NaCl, Baxter, Milan, Italy) or 10% dimethyl sulfoxide. Stock solution of OTA-A (10 mg/mL) was prepared by dissolving the mycotoxin in ethanol protected from direct light. The final OTA concentration was achieved by proper dilution of the stock solution in the respective culture medium. The final ethanol concentration in all working solutions was 0.25 % for each sample.

## Results

### Effects of OTA and HT Cytotoxicity on Cell Viability

**Figure 1:**
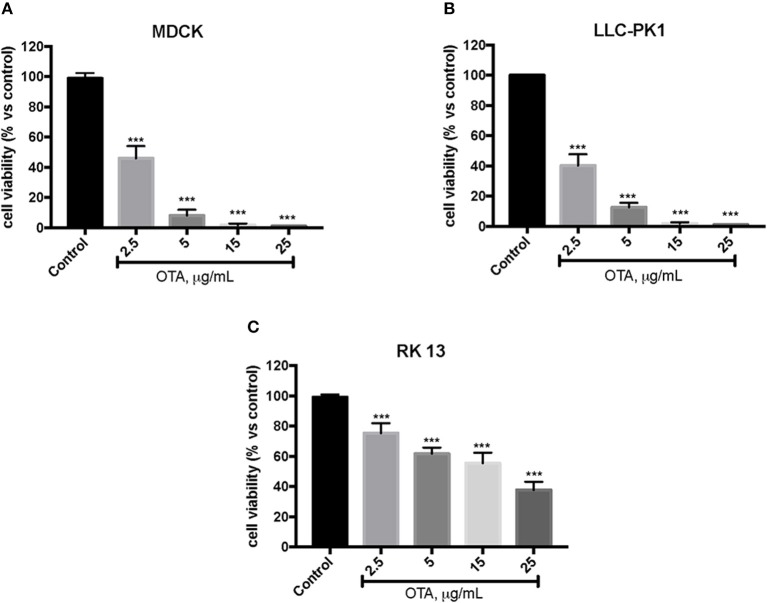
Effect of OTA on cell viability. Cell viability was assessed 24 h after incubation with the indicated concentrations (2.5, 5, 15, and 25 μg/mL) of OTA **(A,B)**. Cell viability was significantly reduced with OTA at 2.5 μg/mL; **(C)** cell viability with OTA at a concentration of 25 μg/mL. Data are representative of at least three independent experiments; ***p < 0.001 vs. Control.

**Figure 2:**
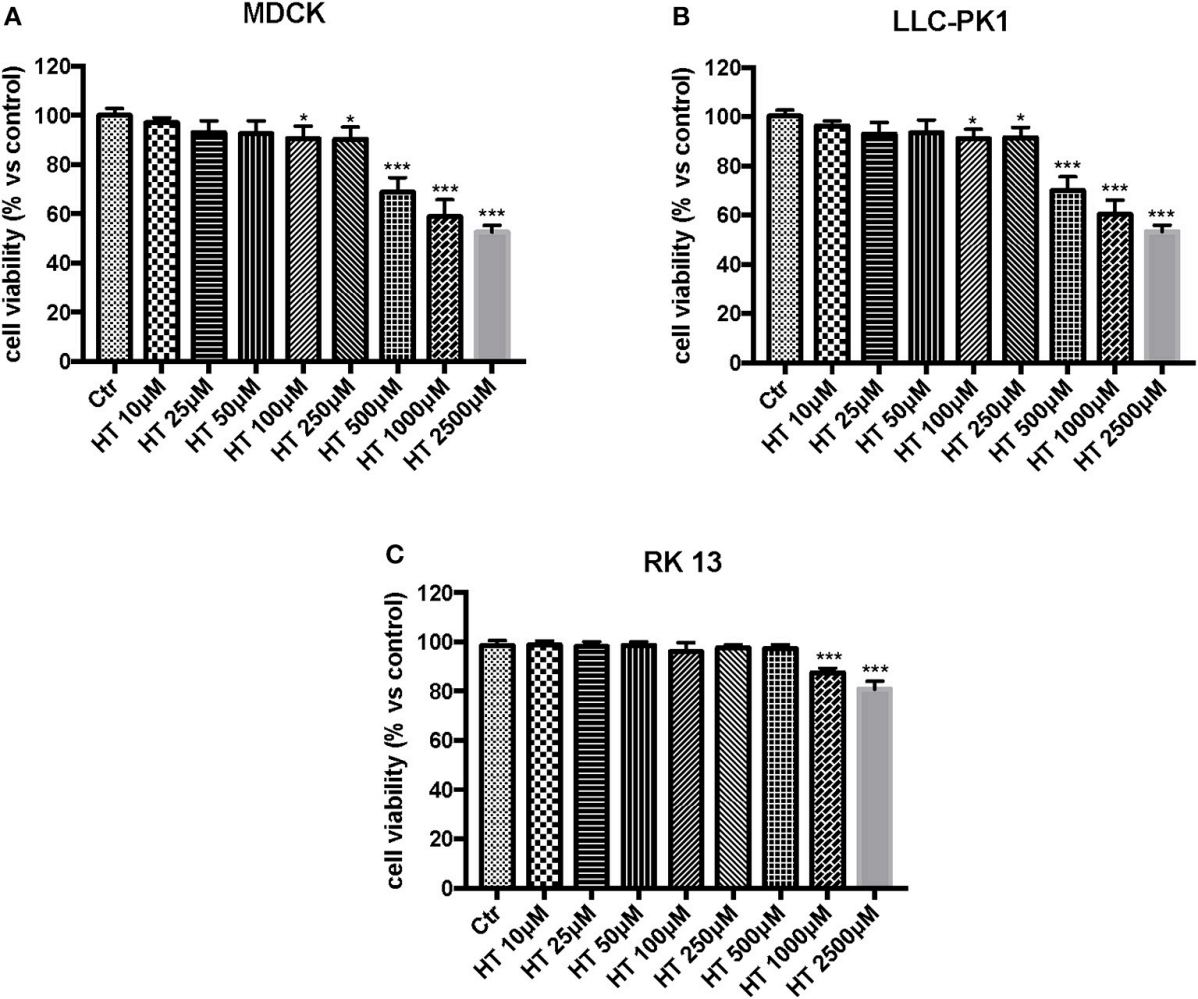
Effect of HT on cell viability. Cell viability was assessed 24 h after treatment with the indicated concentrations (10, 25, 50, 100, 250, 500, 1,000 and 2,500 μM) of HT. **(A,B)** cell viability begins to reduce at the concentration of 500 μM; **(C)** Cell viability was reduced with HT at the concentration of 1,000 μM. Data are representative of at least three independent experiments; *p < 0.05 vs. Control; ***p < 0.001 vs. Control.

**Figure 3:**
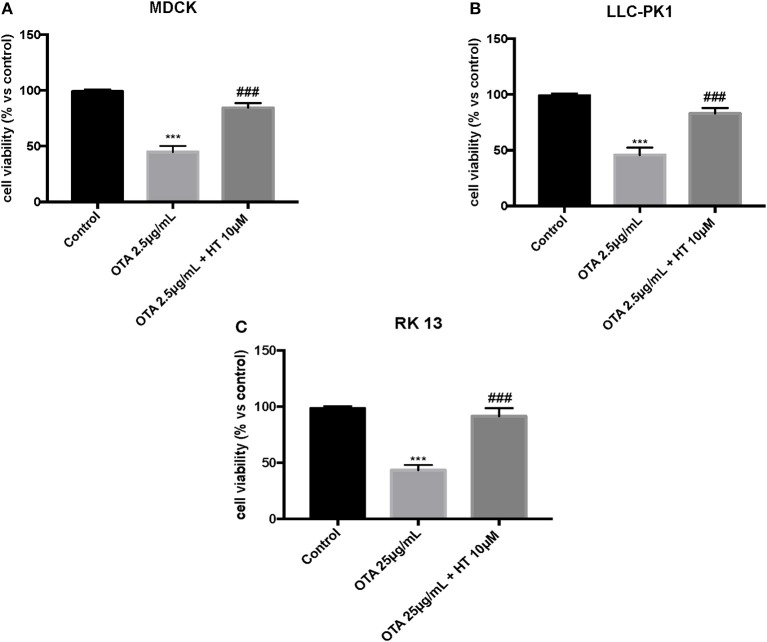
Role of HT on cell viability against OTA **(A,B)**. Incubation of MDCK and LLC-PK1 cells with OTA (2.5 μg/mL) significantly reduced cell viability compared to the control group, whereas pre-treatment with HT at a concentration of 10 μM significantly limited reduction of cell viability **(C)**. Incubation of RK 13 cells with OTA (25 μg/mL) significantly reduced cell viability compared to the control group, whereas pre-treatment with HT at a concentration of 10 μM significantly increased cell viability. Data are representative of at least three independent experiments; ***p < 0.001 vs. Control; ^*###*^*p* < 0.001 vs. OTA.

In the first experiment, we evaluated the effects of diverse OTA concentrations (2.5, 5, 15, 25 μg/mL) on the viability of LLC-PK1, MDCK, and RK 13 cells 24 h after treatment. By MTT assay, we demonstrated that the number of live cells notably diminished with increasing concentrations of OTA. In particular, the OTA concentration of 2.5 μg/mL reduced the number of living MDCK and LLC-PK1 cells to <50% compared to untreated control cells (Figures 1A,B). However, the percent of surviving RK 13 cells was still reasonably high (31%) even after treatment with a 10 times higher OTA concentration (25 μg/mL) (Figure 1C). Therefore, in the following experiments to induce cell damage, we used an OTA concentration of 2.5 μg/mL in MDCK and LLC-PK1 cells, while for RK 13 cells, we used a concentration of 25 μg/mL. In a second experiment, we evaluated the cytotoxicity of HT on MDCK, LLC-PK1, and RK 13 cells. The three different cell lines were incubated with concentrations of HT ranging from 10 to 2,500 μM. In this preliminary study, HT exhibited low cytotoxicity at 10, 25, 50, 100, and 250 μM. However, HT induced a substantial reduction in cell vitality at concentrations of 500 μM and above (Figures 2A–C). Finally, we stimulated cells with OTA (2.5 μg/mL for MDCK and LLC-PK1 cells; 25 μg/mL for RK 13 cells) to induce cellular damage after pretreatment of cells with HT (10 μM). The data obtained revealed a substantial protective effect against OTA-induced cellular damage in cells pretreated with HT at the concentration used (Figures 3A–C).

### Effect of HT on ROS, LDH, and MDA Levels

To better examine the antioxidant capability of HT, we measured the ROS and MDA levels.

**Figure 4:**
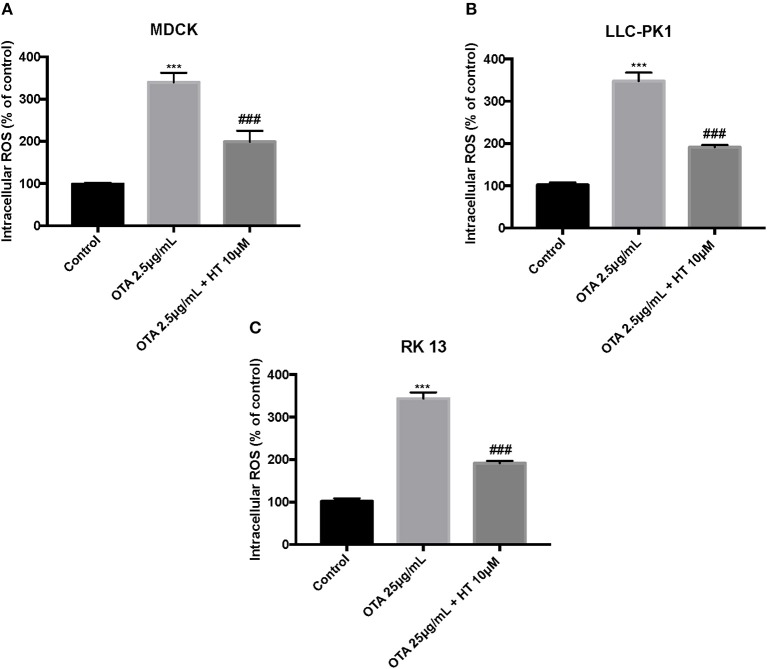
Effect of HT on ROS levels **(A–C)**. We determined the levels of ROS content and observed an increase of ROS levels after OTA stimulation, whereas pre-treatments with HT at a concentration of 10 μM reduced ROS levels. Data are representative of at least three independent experiments; ***p < 0.001 vs. Control; ^*###*^*p* < 0.001 vs. OTA.

**Figure 5:**
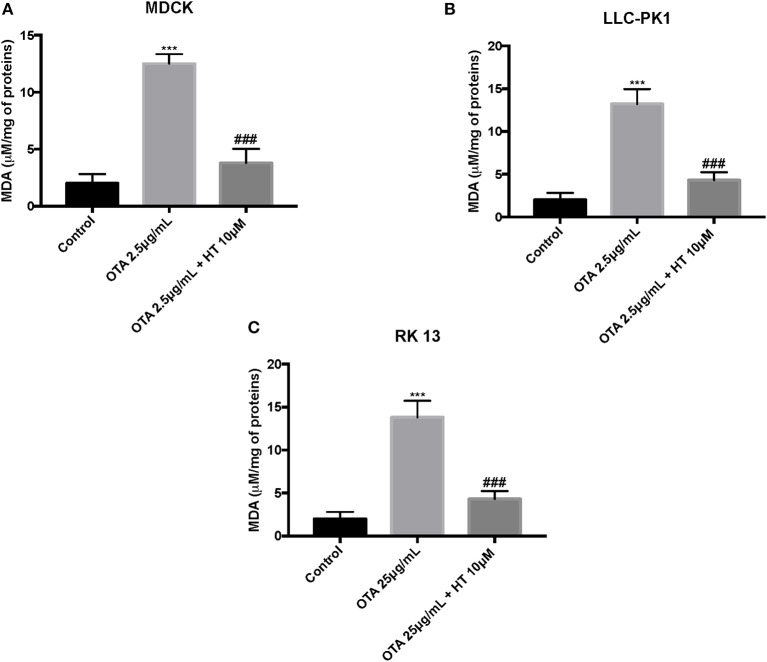
Effect of HT on MDA levels **(A–C)**. We determined the levels of MDA production, and observed an increase of MDA levels after OTA stimulation, whereas pre-treatments with HT at a concentration of 10 μM reduced MDA levels. Data are representative of at least three independent experiments; ***p < 0.001 vs. Control; ^*###*^*p* < 0.001 vs. OTA.

**Figure 6:**
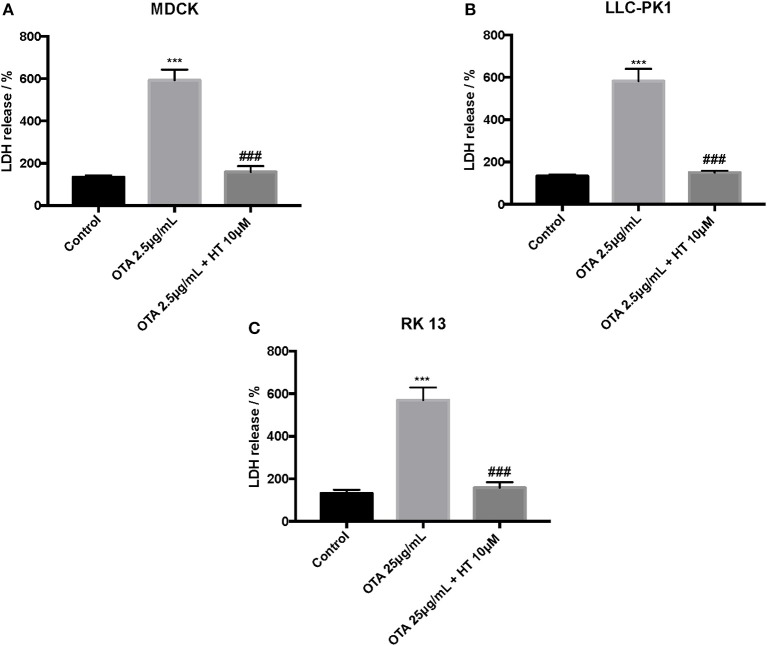
Effect of HT on LDH levels **(A–C)**. We determined the levels of LDH release, and observed an increase of LDH levels after OTA stimulation, whereas pre-treatments with HT at a concentration of 10 μM reduced LDH levels. Data are representative of at least three independent experiments; ***p < 0.001 vs. Control; ^*###*^*p* < 0.001 vs. OTA.

The control group liberated low levels of ROS and MDA, while OTA treatment considerably increased ROS and MDA levels. Pretreatment with HT (10 μM) significantly attenuated ROS (Figures 4A–C) and MDA production (Figures 5A–C). In a successive series of experiments, we assessed LDH release into the culture medium as an indicator of necrotic and/or late apoptotic processes. Our results demonstrated that after treatment with OTA, the release of LDH increased in all three renal cell lines, while pretreatment with HT (10 μM) was able to limit the liberation of LDH into the culture medium (Figures 6A–C).

### Effect of HT on Nitrite Production After OTA-Induced Cell Damage

**Figure 7:**
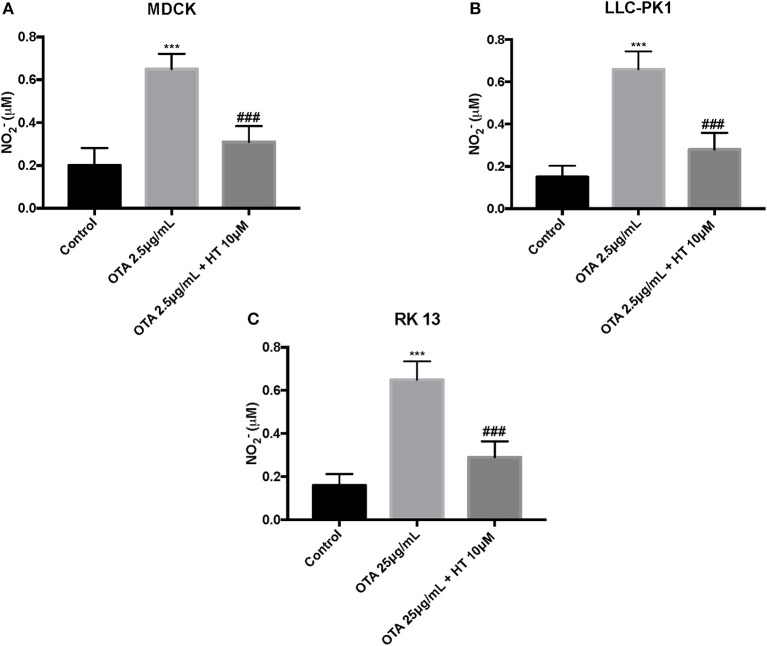
Effect of HT on nitrite levels **(A–C)**. We analyzed the levels of nitrite production and observed an increase in nitrite levels after OTA stimulation, whereas pre-treatments with HT significantly reduced nitrite production. Data are representative of at least three independent experiments; ***p < 0.001 vs. Control; ^*###*^*p* < 0.001 vs. OTA.

We used the Griess assay to measure nitrite release. Our outcomes showed that levels of NO^2−^ production were significantly augmented by OTA. Pretreatment with HT (10 μM) decreased NO release (Figures 7A–C).

### Effect of HT Treatment on Oxidative Stress Marker Alteration After OTA Administration

**Figure 8:**
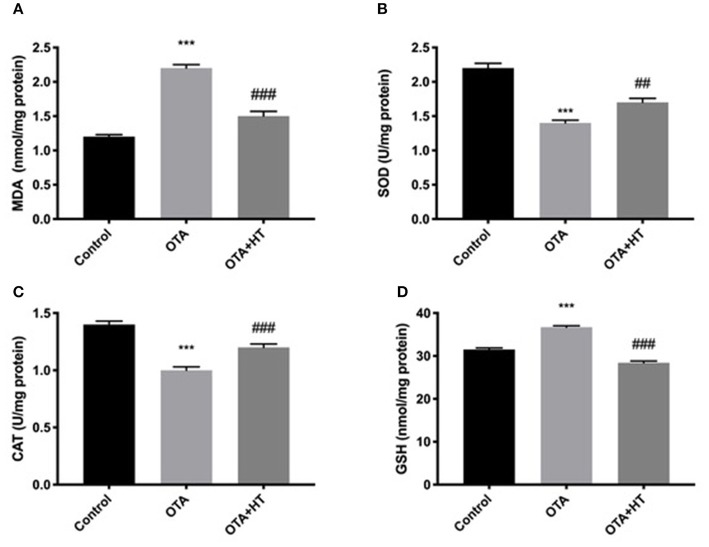
Effect of HT treatment on oxidative stress marker alteration after OTA administration. MDA **(A)**, SOD **(B)**, CAT, **(C)**, and GSH **(D)** were evaluated by ELISA kit. HT administration was able to counteract the increases of MDA and GSH levels and the diminutions of SOD and CAT activity induced by OTA. Data are representative of at least three independent experiments; ***p < 0.001 vs. Control; ^*###*^*p* < 0.001 vs. OTA; ^*##*^*p* < 0.01 vs. OTA.

To evaluate the antioxidant effects of HT, we investigated several indicators of oxidative stress, namely the MDA level, SOD activity, GSH level, and CAT activity. As illustrated in Figure 8A, the results showed a significant decrease in MDA levels in kidney tissue collected from rats treated with HT compared to the OTA group. Additionally, we observed that the activities of SOD (Figure 8B) and CAT (Figure 8C) were significantly increased compared to the OTA group, as was the GSH level (Figure 8D), in the kidneys taken from the HT group.

### Effect of HT Treatment on AST, ALT, and Creatinine After OTA Administration

**Figure 9:**
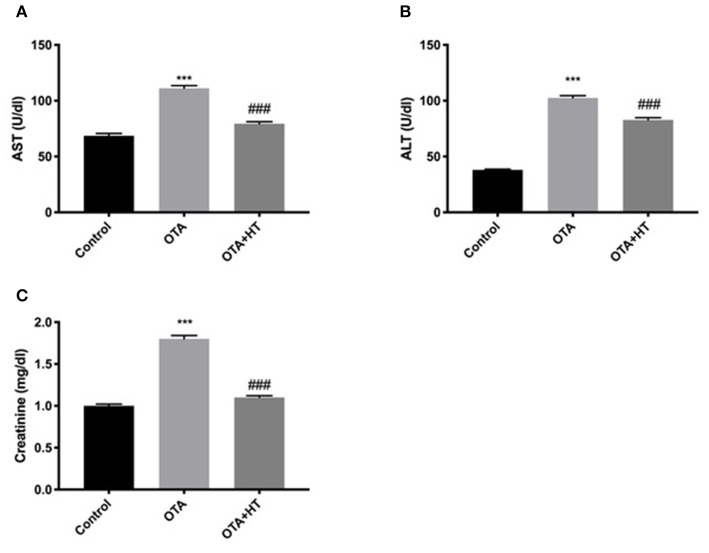
Effect of HT treatment on AST, ALT, and creatinine after OTA administration AST **(A)**, ALT **(B)**, and creatinine **(C)** levels. HT administration was able to counteract the augmentations of AST, ALT, and creatinine levels following OTA induction. Data are representative of at least three independent experiments; ***p < 0.001 vs. Control; ^*###*^*p* < 0.001 vs OTA.

Additionally, we assessed the effect of HT during OTA-induced toxicity. As shown in Figure 9, we found a substantial increase in AST (Figure 9A), ALT (Figure 9B), and creatinine (Figure 9C) levels after OTA administration. HT treatment was able to significantly counteract these augmentations.

### Effect of HT Administration on Kidney Fibrosis and TGFβ-1 and α-sma Expressions

**Figure 10:**
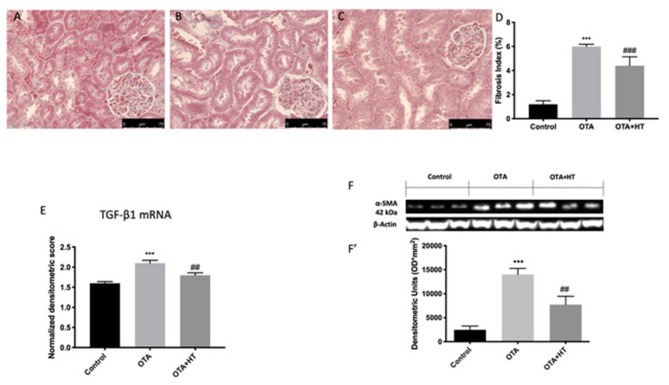
Effect of HT administration on kidney fibrosis, TGFβ-1, and α-sma expressions Masson's Trichrome of control **(A)**, OTA **(B)**, and HT **(C)**; fibrosis index **(D)**, TGFβ-1 **(E)**, and α-sma **(F,F′)** expressions. HT treatment was able to decrease kidney fibrosis and TGFβ-1 and α-sma expressions. Data are representative of at least three independent experiments; ***p < 0.001 vs. Control; ^*###*^*p* < 0.001 vs. OTA; ^*##*^*p* < 0.01 vs. OTA.

An increase in collagen secretion can be an indicator of the induction of fibrosis, which reportedly is one outcome of OTA exposure (31). For this reason, we evaluated kidney fibrosis by Masson's trichrome staining. In the OTA group, we found an increase in blue color, indicating an increase in collagen fibers (Figure 10B, see fibrosis index 3D) compared to the control group (Figure 10A, see fibrosis index 10D). HT diminished collagen accumulation (Figure 10C, see fibrosis index Figure 10D). To confirm our results, we evaluated TGF-β1 and α-sma, which are generally considered mediators of fibrotic diseases (22, 32). We found that following OTA administration, there was an increase in the expression of both TGF-β1 and α-sma (Figure 10F see densitometric analysis F') compared to the control group. On the other hand, HT was able to restore TGF-β1 levels (Figure 10E), as well as α-sma expression (Figure 10F see densitometric analysis F').

### Effect of HT Administration on Kidney Histology

**Figure 11:**
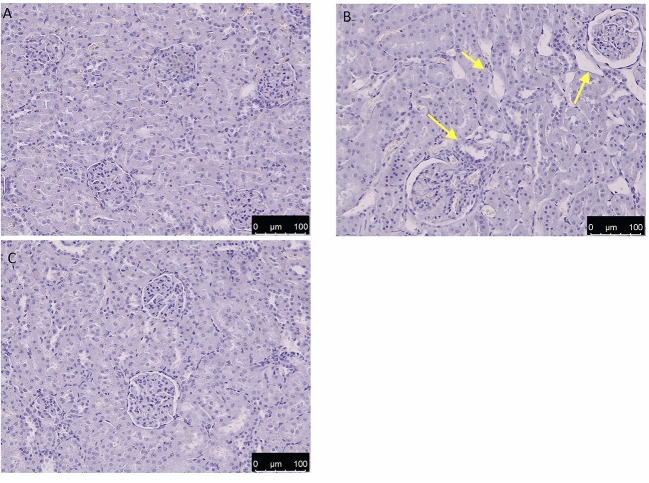
Effect of HT administration on kidney histology H/E of control **(A)**, OTA **(B)**, and HT **(C)**. HT group showed a decrease in OTA-induced histological alteration, which is highlighted by yellow arrows in the OTA group. No alteration was observed in the control group.

As shown in Figure 11B, after OTA administration, the kidney sections showed clear degenerative lesions when compared to control group Figure 11A, mainly located in the inner part of the cortex. Indeed, as shown in Figure 11C, administration of HT led to a significant increase in the severity of the damage.

## Discussion

In the present study, MDCK, LLC-PK1, and RK 13 cells and male Sprague–Dawley rats were used as models for studying the renal toxicity induced by OTA. OTA has recently been cited as an important contaminant found not only in plant foods but also in products of animal origin. It appears in the latter as a result of its accumulation in muscles, organs, and offal (the kidney and liver, in particular). It is also frequently employed in high quantities in the pet food sector, in particular for the formulation of wet products (33). Several studies have shown that OTA has chronic harmful effects in mammals even at low concentrations (22, 34, 35). Repeated exposure to OTA causes impaired renal function and morphology as well as an amplified incidence of renal adenoma and carcinoma due to the increase in oxidative stress induced by OTA. Considering the nephrotoxic potential of OTA, in the first set of experiments, we observed the effect of this mycotoxin on cell viability using the MTT test. The cell viability assays showed significant cytotoxicity of OTA (2.5 μg/mL) in MDCK and LLC-PK1 cells, while we observed a notable decrease in the number of living RK 13 cells at a concentration of 25 μg/mL. Currently, it is well-known from epidemiological studies that a dietary intake of foods rich in antioxidants, such as phenolic compounds, is related to a reduced risk of renal dysfunction (17). Among the major phenols contained in extra virgin olive oil, HT has antioxidant powers that are well-known. The driving idea of this study was to investigate the potential protective effects of HT against OTA-induced injury. First, the three different renal cell lines were examined with an *in vivo* study. Furthermore, before evaluating the protective effects of HT on the different renal cell lines, we verified the cellular cytotoxicity of this phenolic compound using the MTT assay. Our results showed that HT began to be cytotoxic at a concentration of 250 μM, which may be due to the pro-apoptotic action of HT as its concentration increases, as has been seen in several studies. However, this effect of HT has not been fully clarified (36). Therefore, in a subsequent cell viability test, we verified the ability of HT (10 μM) to reduce the cellular mortality induced by OTA. The loss of cell viability after OTA exposure might be due to the impairment of cell membrane integrity induced by high ROS production, leading to LDH leakage. To confirm this hypothesis, we evaluated the ROS and LDH levels after OTA treatment. Our results showed an increase of ROS and LDH in renal cells treated with OTA, while the pretreatment of renal cells with HT limited the release of LDH into the culture medium and also the ROS levels. Moreover, particularly in cell membranes, during lipid peroxidation, a number of secondary molecules are produced, such as MDA, which is a marker for cell oxidation. Therefore, to further confirm the antioxidant capabilities of HT, we measured the MDA levels. Our results demonstrated that OTA treatment induced a major increase in the levels of MDA. Previous findings suggest that HT exerts a marked protective effect against oxidative damage, especially in renal cells, in particular preserving the membrane lipids due to the scavenger action of HT on ROS. Our results show that HT preserves the integrity of biological membranes from negative oxidative processes caused by free radicals in kidney cells. We also wanted to assess the effect of HT on nitrosative stress. In this regard, we measured the levels of nitrite production. Our data demonstrated that pre-treatment with HT was able to reduce the levels of NO, which increased in cells treated only with OTA. We also evaluated the effect of HT treatment on the renal toxicity induced by OTA administration *in vivo*. Consistent with the *in vitro* analysis, rats administered OTA displayed increased levels of the lipid peroxidative marker malondialdehyde (MDA) and reduced levels of non-enzymatic antioxidants, such as SOD, GSH, and CAT, compared with controls. HT treatment showed a protective effect on OTA-induced oxidative damage. To assess the influence of HT treatment on the liver function of OTA-treated rats, the enzymes aspartate-aminotransferase (AST) and alanine-aminotransferase (ALT) were evaluated. OTA administration induced an increase in the serum hepatic biomarkers AST and ALT compared with the normal control, while treatment with HT significantly reduced the levels compared to the OTA group. This gives an indication of the important protective effect of HT, even if the levels of these enzymes have not been restored to baseline. This may depend on the type of liver damage caused by OTA; in fact, it has recently been shown that OTA is also capable of inducing liver inflammation (37). OTA has been seen to be directly linked to kidney diseases, and in fact, we found significantly increased creatinine levels in OTA-treated animals. HT treatment was able to reduce creatinine levels to normal values. OTA-induced nephropathy is characterized by the growth of extracellular matrix (ECM) components, leading to tubulointerstitial fibrosis and glomerulosclerosis (38). Moreover, OTA-treated animals showed increased levels of TGF-β1, one of the most important mediators of chronic renal fibrosis (39), and α-sma, the increase in which is considered a key event in tubular epithelial-mesenchymal transdifferentiation (EMT) (40). HT treatment reduced collagen deposition and TGF-β1 and α-sma accumulation in the kidneys of OTA-treated rats. This effect of HT is more evident for TGF-β1; in fact, the expression of TGF-β1 in HT treated groups is comparable with the control group. Additionally, from a histological point of view, HT treatment improved the degenerative lesions of the kidney induced by OTA administration.

## Conclusions

The outcomes of this study demonstrate the efficacy of HT in counteracting OTA-induced oxidative damage in MDCK, LLC-PK1, and RK 13 cells and against OTA-induced nephrotoxicity in rats. In addition, the administration of HT can reduce the abnormalities of liver and kidney biomarkers induced by OTA administration. Our results encourage the hypothesis that olive oil phenolics may support decreased kidney exposure to the development of oxidative stress-related renal failure.

## Data Availability Statement

The datasets generated and/or analyzed for the present study are available from the corresponding author on reasonable request.

## Ethics Statement

The animal study was reviewed and approved by The University of Messina Review Board for the care of animals.

## Author Contributions

RC, EP, and RS: contribution to conception and design, interpretation of data, and drafting of the manuscript. EG, RF, MC, DI, and CD: acquisition of data and analysis. LC, SC, and RD: revising the manuscript critically. All authors have read and approved the final manuscript.

### Conflict of Interest

The authors declare that the research was conducted in the absence of any commercial or financial relationships that could be construed as a potential conflict of interest.
